# Spontaneous retroperitoneal hemorrhage caused by rupture of an ovarian artery aneurysm: a case report and review of the literature

**DOI:** 10.1186/s13256-015-0553-4

**Published:** 2015-04-18

**Authors:** Masafumi Toyoshima, Takako Kudo, Saori Igeta, Hiromitsu Makino, Yuta Momono, Takashi Shima, Rui Matsuura, Nobuko Ishigaki, Kozo Akagi, Yoichi Takeyama, Hideki Iwahashi, Hiroya Rikimaru, Akihiro Sato, Kosuke Yoshinaga

**Affiliations:** 1 Department of Obstetrics and Gynecology, Sendai Medical Center, National Hospital Organization, 2-8-8, Miyagino, Miyagino-ku, Sendai, Miyagi 983-8520 Japan; 2 Department of Radiology, Sendai Medical Center, National Hospital Organization, Sendai, Japan; 3 Department of Obstetrics and Gynecology, Self-Defense Force Sendai Hospital, Sendai, Japan

**Keywords:** Aneurysm, Multigravida, N-butyl-2-cyanoacrylate, Ovarian artery, Retroperitoneal hematoma, Spontaneous rupture, Transcatheter arterial embolization

## Abstract

**Introduction:**

Spontaneous rupture of an ovarian artery aneurysm is extremely rare. Although a majority of these cases have been associated with pregnancy, there have been recent reports and reviews of rare cases that were not directly associated with pregnancy. Transcatheter arterial embolization is considered to be an alternative therapy to surgery.

**Case presentation:**

A 44-year-old Japanese woman, gravida 3 para 3, presented to our emergency room complaining of intermittent right flank pain. She had undergone a cesarean section 2 years previously, and had no history of abdominal trauma. On admission, her blood pressure was 115/78 mmHg, pulse 70 beats per minute, and hemoglobin concentration 9.8 g/dL. Abdominal ultrasonography and contrast-enhanced dynamic computed tomography revealed a large retroperitoneal hematoma. Findings on three-dimensional computed tomography angiography suggested ruptured aneurysm of her right ovarian artery. A selective right ovarian artery angiogram revealed a tortuous aneurysm. Transcatheter arterial embolization using N-butyl-2-cyanoacrylate was performed. The aneurysm was successfully embolized, and her course after embolization was uneventful. She has remained symptom-free during 3 months of follow-up.

**Conclusions:**

This was a very rare case of a patient who had a retroperitoneal hemorrhage originating from an ovarian artery aneurysm. A review of published case reports found that contrast-enhanced computed tomography with reconstruction images is an excellent imaging tool. Diagnostic angiography and subsequent transcatheter arterial embolization are thought to be very effective for this condition.

## Introduction

Spontaneous rupture of an ovarian artery aneurysm is extremely rare; only 25 cases have been reported in the English literature [1-24]. Although a majority of these cases were related to pregnancy and occurred during the peripartum or postpartum period, there have been recent reports and reviews of rare cases that were not directly associated with pregnancy [7,14,16,18-20,22]. Rupture of an ovarian artery aneurysm leads to retroperitoneal hemorrhage and can be a life-threatening event. According to previously reported cases, life-saving treatment of a ruptured ovarian artery consists of surgery that includes ligation of the artery proximal and distal to the site of rupture. However, since the first report by King [9], transcatheter arterial embolization (TAE) has emerged as an alternative therapy for patients who are hemodynamically stable. Here, we report the case of a patient with spontaneous retroperitoneal hemorrhage associated with rupture of an ovarian artery aneurysm who was successfully treated using TAE only. We also review the published case reports on this rarely occurring condition.

## Case presentation

A 44-year-old Japanese woman, gravida 3 para 3, who had undergone cesarean section 2 years previously, presented to our emergency room with a 2-day history of intermittent right flank pain. She had no fever, nausea, vomiting, diarrhea, or cough. She had no history of abdominal trauma, and her past medical history and family history were not significant. She did not have hypertension or cardiovascular disease, and had not taken any anticoagulants. Her bowel and urinary habits were normal, and her menstrual periods were regular. Her last menstrual period had begun 2 days before the onset of right flank pain. On admission, her blood pressure was 115/78 mmHg, pulse 70 beats per minute, body temperature 36°C, and blood oxygen saturation 100%. She was found to be somewhat anemic, with a hemoglobin concentration of 9.8 g/dL and hematocrit of 28.2%. Her white blood cell count was elevated (13,900/mm^3^), and a urine pregnancy test was negative.

**Figure 1:**
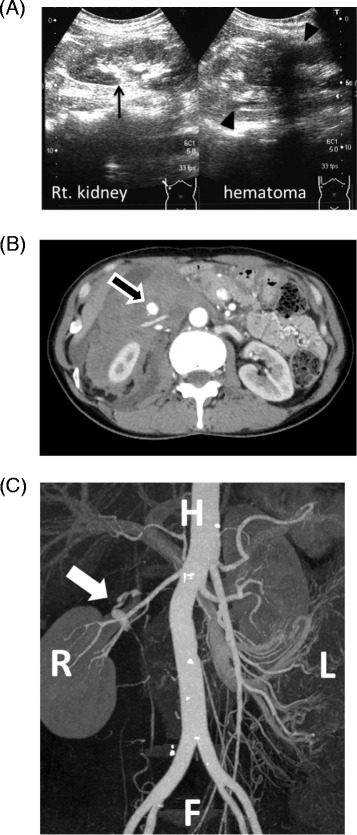
Imaging tests for retroperitoneal hematoma. **(A)** Abdominal ultrasonography demonstrated a normal right kidney (left side, arrow) and a large hematoma in the retroperitoneum. A high-echoic lesion can be seen surrounding the right kidney (right side, arrowhead). **(B)** Arterial phase contrast-enhanced computed tomography image. A bright round structure (arrow) can be seen in the right retroperitoneal hematoma. **(C)** Three-dimensional computed tomography angiogram of the abdomen revealed a right ovarian artery aneurysm (arrow) overriding the right renal artery. Abbreviations: L, left; R, right; Rt., right; H, head, F, foot.

**Figure 2:**
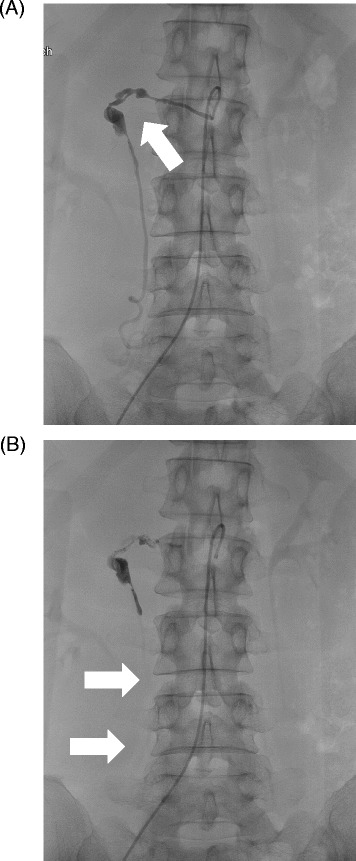
Angiograms before and after transcatheter arterial embolization. **(A)** Selective angiogram of the right ovarian artery showing several aneurysms (arrow) located near the origin from the aorta. **(B)** Angiogram obtained after N-butyl-2-cyanoacrylate embolization showing successful embolization of the aneurysm. Note that the distal tortuous section of the right ovarian artery disappear (arrows).

On physical examination, her abdomen was diffusely tender without muscle guarding. A pelvic examination revealed a small amount of menstrual discharge and a normal uterus and bilateral adnexae. Abdominal ultrasonography demonstrated a large retroperitoneal hematoma surrounding her right kidney (Figure 1A). Emergent abdominal and pelvic computed tomography (CT) was performed. Contrast-enhanced dynamic CT revealed a large retroperitoneal hematoma surrounding her right kidney with an enhancing round structure in the center of the hematoma in the arterial phase (Figure 1B). Although extravasation in the venous phase was not clear, findings on three-dimensional CT angiography were suggestive of a retroperitoneal hematoma due to rupture of an aneurysm of her right ovarian artery (Figure 1C), and no other responsible lesion was seen. A transfemoral angiography was performed for arterial embolization under a clinical diagnosis of bleeding from a right ovarian artery aneurysm. A selective angiogram of her right ovarian artery revealed a tortuous aneurysm near its origin from the aorta without obvious active extravasation (Figure 2A). A 2.1-Fr microcatheter (Tangent™; Boston Scientific, USA) was advanced into the orifice of the aneurysm, and 1mL of 16.7% N-butyl-2-cyanoacrylate (NBCA) diluted in iodized oil (Lipiodol®; Guerbet Japan, Tokyo, Japan) was manually injected beyond the distal site of the aneurysm. A postembolization angiogram showed complete occlusion of the vessel (Figure 2B). No other aneurysm was found on three-dimensional CT and angiography.

One day after TAE, CT was performed, which showed that the hematoma had decreased in size, and there was no sign of extravasation. In addition, her hemoglobin and hematocrit were found to have dropped to 7.9 g/dL and 24.1%, respectively. She was administered iron for 4 days, with a subsequent increase in hemoglobin and hematocrit to 8.9g/dL and 25.6%, respectively. No other surgical intervention was needed, and her course after embolization was uneventful. She was discharged on the fifth hospital day, and has remained symptom-free during 3 months of follow-up.

## Discussion

A retroperitoneal hematoma can be a life-threatening event, as well as a surgical emergency. Abdominal trauma, including iatrogenic injuries from surgical interventions such as inferior vena cava filter placement and arterial puncture, is the most common cause of retroperitoneal hemorrhage. In addition, a ruptured aortic or renal artery aneurysm, retroperitoneal tumors, and clotting disorders have been mentioned as the main causes of retroperitoneal hematoma [25].

The most common presenting symptom of retroperitoneal hematoma is acute abdominal or flank pain accompanied by clinical signs associated with bleeding. However, the clinical picture is often nonspecific, and the ruling out of acute abdomen, ureteral calculus, and pyelonephritis is needed. In addition, pregnant patients in the third trimester can be misdiagnosed with placental abruption or uterine rupture if they do not undergo an adequate imaging examination such as contrast-enhanced CT, although pregnancy is a relative contraindication to this procedure. The most common arterial sites for ruptured aneurysm in order of frequency during pregnancy are the aorta, cerebral arteries, splenic artery, renal artery, coronary artery, and ovarian artery [26].

Based on a MEDLINE search of the English language literature from 1963 to 2014, to the best of our knowledge, only 25 cases of spontaneous rupture of an ovarian artery aneurysm have been reported [1-24]. Of these reported cases, 18 cases (72%) were associated with pregnancy (Table 1), and seven cases (28%) were not directly related to pregnancy (Table 2). The patients whose pregnancy and delivery history were available were all multigravida. The ages of the patients with pregnancy-related ruptured ovarian aneurysm ranged from 23 to 39 years (mean 33.6 and median 35 years), and the ages of the other patients ranged from 45 to 69 years (mean 51.5 and median 49.5 years). There seemed to be no difference regarding the side of the body where the ruptured aneurysm occurred. Among pregnancy-related patients, the aneurysm occurred on the left in eight and right in 10 cases; and among the other patients, on the left in five and right in three cases, including the present case. The majority of ruptures occurred during the postpartum period, which accounted for 14 out of 18 cases (78%). The onset of rupture occurred during the third trimester in two patients, who underwent cesarean section followed by ovarian artery ligation.

**Table 1:** **Reported 18 cases of pregnancy-related spontaneous rupture of the ovarian artery**

**Age (years)**	**Gravida/para**	**Side**	**Onset**	**Treatment**	**Author, year**
29	G4P4	L	2d postpartum	Laparotomy	Caillouette and Owen, 1963 [1]
35	G6P3	L	4d postpartum	Laparotomy	Tsoutsoplides, 1967 [2]
38	G6P6	R	During delivery	Laparotomy	Riley, 1975 [3]
32	G3P3	L	4d postpartum	Laparotomy	Burnett and Carfrae, 1976 [4]
35	G3P3	R	4d postpartum	Laparotomy	Same as above
26	G5P4	R	1d postpartum	Laparotomy	Jafari and Saleh, 1977 [5]
23	N/A	R	1m postpartum	Laparotomy	Mojab and Rodriguez, 1977 [6]
31	G4P3	R	39w of gestation	Laparotomy (caesarean section, ovarian artery ligation)	Høgdall *et al*., 1989 [8]
36	G5P5	R	4d postpartum	TAE	King, 1990 [9]
38	G3P2	R	During delivery	Laparotomy	Belfort *et al*., 1993 [10]
38	G3P2	R	4d postpartum	TAE→ lomboscopic drainage	Guillem *et al.*, 1999 [11]
38	G12P11	R	3d postpartum	Laparotomy	Blachar *et al*., 2000 [12]
37	P4	L	39w of gestation	Laparotomy (caesarean section, ovarian artery ligation)	Panoskaltsis *et al*., 2000 [13]
30	G5P5	L	5h postpartum	Laparotomy	Kale *et al*., 2005 [15]
39	G5P4	R	5d postpartum	TAE	Poilblanc *et al*., 2008 [17]
32	P4	L	2d postpartum	Laparotomy→TAE	Mohammed *et al*., 2011 [21]
37	G4P4	L	4d postpartum	TAE	Wakimoto *et al*., 2014 [23]
31	G6P4	L	2d postpartum	TAE	Sakaguchi *et al*., 2014 [24]

Abbreviations: d, day(s); h, hour(s); L, left; m, month; N/A, not available; R, right; TAE, transcatheter arterial embolization; w, week(s).

**Table 2:** **Present case and seven reported cases of pregnancy-unrelated spontaneous rupture of the ovarian artery**

**Age (years)**	**Gravida/Para**	**Location**	**Onset**	**Treatment**	**Author, year**
45	G6P5	L	Follicular phase	Laparotomy	Siu *et al*., 1986 [7]
53	G1P1	L	Postmenopause	Laparotomy	Manabe *et al*., 2002 [14]
55	G2P2	R	Postmenopause	TAE	Nakajo *et al*., 2005 [16]
46	G3P2	L	2d of menstruation	TAE(failed)→ Laparotomy	Chao and Chen, 2009 [18]
69	G3P3	L	Postmenopause	TAE	Kirk *et al.*, 2009 [19]
48	G2P2	L	2d of menstruation	TAE(failed)→ Laparotomy	Tsai and Lien, 2009 [20]
51	G3P3	R	Postmenopause	Laparotomy	Kodaira *et al*., 2014 [22]
**45**	**G3P3**	**R**	**3d of menstruation**	**TAE**	**Present case**

Abbreviations: d, day(s); L, left; R, right; TAE, transcatheter arterial embolization.

Because a ruptured ovarian artery aneurysm mostly occurs in women of high parity, the repeated hemodynamic and endocrine changes during pregnancy are thought to be the cause of arterial alterations that may lead to new aneurysm formation and/or weakening of pre-existing aneurysms [4,26]. In addition to the physiologic changes of pregnancy, hypertension might be a risk factor for rupture of ovarian artery aneurysm [18].

In earlier reported cases, the diagnosis was always obtained by exploratory laparotomy. Since Blachar *et al.* [12] first reported on the use of contrast-enhanced CT with reconstruction images, this imaging modality has been found highly effective for the preoperative diagnosis of ruptured ovarian artery aneurysm.

Of 17 cases published in 1990 or later, TAE was attempted as a treatment for ruptured ovarian artery aneurysm in 11 cases (64.7%), and the procedure was successful in nine cases, including ours. Because these patients had good outcomes after a minimally invasive procedure, TAE is currently considered to be the treatment of choice. The following embolic agents have been used in the previous reports; microcoils in five cases [9,11,17,19,21], gelatin sponge particles (GSPs) in one case [24], a combination of microcoils and GSP in one case [16], and NBCA was recently used in one case [23]. Our patient is the second case whose ruptured ovarian artery aneurysm was successfully treated using NBCA.

The mechanism of embolization by microcoil and GSP involves thrombin formation, which requires normal clotting function. However, NBCA occludes blood vessels because it polymerizes on contact with plasma, and therefore this method does not depend on the patient’s hemostatic capacity. Yonemitsu *et al.* evaluated the outcomes of TAE performed using microcoils, GSP, and NBCA in the setting of coagulopathy. They reported that the rate of primary hemostasis was significantly higher in patients undergoing NBCA compared to GSP, and the mean treatment time was significantly shorter for the NBCA procedure compared to the microcoil procedure [27]. Therefore, it is reasonable to consider NBCA as the embolic agent of choice for ruptured ovarian artery aneurysm, especially for critical patients in shock, who tend to have clotting problems due to coagulopathy. Because most patients with ovarian artery aneurysm are asymptomatic and rupture is an uncommon event, this condition may be underdiagnosed, and the risk factors for rupture have not yet been studied in depth. Additional case reports may help clarify the nature of ovarian artery aneurysms.

## Conclusions

We presented a very rare case of a patient who had a retroperitoneal hemorrhage that originated from an ovarian artery aneurysm. Contrast-enhanced CT with reconstruction images has been found to be an excellent imaging tool. Diagnostic angiography combined with subsequent TAE during the same imaging session is thought to be a useful and highly effective alternative therapeutic procedure for this condition. Because ovarian arteries are potential sources of pelvic hemorrhage, these vessels should be routinely studied when imaging demonstrates the presence of a retroperitoneal hematoma, especially in multiparous women, whether or not they are pregnant.

## Consent

Written informed consent was obtained from the patient for publication of this case report and accompanying images. A copy of the written consent is available for review by the Editor-in-Chief of this journal.

## Abbreviations

CT: Computed tomography
GSP: Gelatin sponge particle
NBCA: N-butyl-2-cyanoacrylate
TAE: Transcatheter arterial embolization
